# Lack of association between dietary fructose and hyperuricemia risk in adults

**DOI:** 10.1186/1743-7075-7-16

**Published:** 2010-03-01

**Authors:** Sam Z Sun, Brent D Flickinger, Patricia S Williamson-Hughes, Mark W Empie

**Affiliations:** 1 Office of Compliance and Ethics, Archer Daniels Midland Company, 1001 North Brush College Road, Decatur, Illinois 62521, USA

## Abstract

**Background:**

High serum uric acid concentration (hyperuricemia) has been studied for its relationship with multiple adverse health outcomes, such as metabolic syndrome. Intervention studies have produced inconsistent outcomes for the relationship between fructose intake and serum uric acid concentration.

**Methods:**

The association of dietary fructose intake with hyperuricemia risk in adults was examined using logistic regression and U.S. NHANES 1999-2004 databases. A total of 9,384 subjects, between the ages 20 and 80 years, without diabetes, cancer, or heart disease, were included.

**Results:**

The highest added or total fructose intake (quartiles by grams or % energy) was not associated with an increase of hyperuricemia risk compared to the lowest intake with or without adjustment (odds ratios = 0.515-0.992). The associations of alcohol and fiber intakes with the risk were also determined. Compared to the lowest intake, the highest alcohol intake was associated with increased mean serum uric acid concentration (up to 16%, *P *< 0.001) and hyperuricemia risk (odds ratios = 1.658-1.829, *P *= 0.057- < 0.001); the highest fiber intake was correlated with decreases of uric acid concentration (up to 7.5%, *P *< 0.002) and lower risk (odds ratios = 0.448-0.478, *P *= 0.001- < 0.001). Adults who were over 50 y old, male, or obese had significantly greater risk.

**Conclusions:**

The data show that increased dietary fructose intake was not associated with increased hyperuricemia risk; while increased dietary alcohol intake was significantly associated with increased hyperuricemia risk; and increased fiber intake was significantly associated with decreased hyperuricemia risk. These data further suggest a potential effect of fructose consumption in an ordinary diet on serum uric acid differs from results found in some short-term studies using atypical exposure and/or levels of fructose administration.

## Introduction

Uric acid or urate is a metabolite of purine or purine-containing compounds and is considered to be one of the major endogenous antioxidants. In humans, urate is excreted as an end product while in other species it is further metabolized to allantoin. Recent studies have indicated that high serum uric acid concentration may be an etiological factor for diabetes, hypertension, metabolic syndrome, and heart disease [1-8]. In addition to intake of purine-rich foods, such as certain kinds of meat or seafood, fructose or fructose-containing foods have been linked to increases in serum uric acid concentration as a result of excessive fructose phosphorylation and rapid expenditure of adenosine triphosphate (ATP) over-yielding purines. A controversial hypothesis has been recently suggested by Johnson et al [9], purporting that high fructose intake may increase serum uric acid concentration, leading to the development of diabetes. Early intervention studies conducted by Macdonald [10], Emmerson [11], and Fox [12] noted that fructose given experimentally by ingestion or infusion could induce acute increases of serum uric acid concentrations. However, other studies reported by Crapo [13], Huttunen [14], Turner [15], Curreri [16], Osei [17,18], Anderson [19], Koh [20], and Grigoresco [21] did not observe that fructose influenced serum uric acid concentrations. Narins [22] et al. reported a mixed effect of fructose on serum uric acid concentration.

The generalization of an outcome from an experimental nutrition trial to an individual's ordinary food consumption is complicated. The interpretation of a slight, though statistically significant, change in a biochemical measurement, such as serum uric acid, into clinical relevance can be difficult especially when the change is within a normal clinical range. Very recently, a review by Livesey [23] indicated that: "Intervention studies in humans often use fructose at doses that are excessive compared with amounts generally eaten by adults; such are not interpretable for purposes of public health policy in adult population."

In view of limitations and inconsistent outcomes of fructose-urate connection from intervention studies, we examined the relationship between dietary fructose intakes and risk of hyperuricemia occurrence in the U.S. adult population with the added assessments of alcohol and fiber intakes. Alcohol is a known factor to raise uric acid levels, and fruits and vegetables (which contain significant levels of fiber) are associated with decreasing uric acid levels [24]. Therefore, alcohol and fiber intakes were examined as indicators of model appropriateness. It is expected that this work, utilizing a large population-based approach and data obtained under real-life setting, can provide more understanding of the potential association between dietary fructose and uric acid concentration under typical living conditions.

## Methods

### Data and subjects

Data of dietary intakes, demographics, health status, and blood chemistries were extracted from databases of the U.S. Center for Disease Control (CDC), National Health and Nutrition Examination Survey (NHANES) 1999-2000, NHANES 2001-02, and NHANES 2003-04 (publically available from http://www.cdc.gov/nchs/nhanes.htm). NHANES is a program of studies designed to assess the health and nutritional status of adults and children in the United States. The study protocol of NHANES was approved by Research Ethics Review Board of CDC National Center for Health Statistics, and documented consent was obtained from participants. Adults (N = 9,384) aged 20-80 y, without diabetes, cancer, and/or heart disease were included in this analysis. Young people were excluded because they are not permitted by law to consume alcohol in the U.S. Among the dietary intake variables assessed (energy, protein, total fat, fructose, alcohol, fiber, vitamin C, and caffeine), the intakes of fructose, alcohol, and fiber were specifically emphasized in this work. For the dietary intake data, values that did not meet the criteria of reliable recall status were not used. Demographic factors used in the analysis included gender, age, race, body weight status, and education level.

### Term definition and subject categorization

The key outcome variable assessed in this work was the risk of hyperuricemia. Based on CDC's definition in NHANES 2001-08, the normal medical reference values of serum uric acid are 3.6-8.4 mg/dL for men and 2.9-7.5 mg/dL for women (214-500 and 172-449 μmol/L, 1 mg/dL = 59.48 μmol/L), respectively [25]. Thus, we defined a man as hyperuricemic if his uric acid concentration is > 8.4 mg/dL, and a woman as hyperuricemic if her uric acid concentration is > 7.5 mg/dL. Some previous studies have defined hyperuricemia as serum uric acid concentration > 7 mg/dL for men and > 6 mg/dL for women. However, we chose to utilize the relevant CDC's reference in this work because it is a nationally recognized source. Dietary intake variables, which may potentially influence the study outcome, included energy (kcal, 1 kcal = 4.184 kJ), protein, total fat (% of daily energy), fiber (grams/1000 kcal), vitamin C, caffeine, added unbound fructose (mono-saccharide fructose), all added fructose (unbound + bound), and total fructose (added and naturally-occurring unbound fructose). These variables were assigned as quartile intakes (1 to 4) based on their 25^th^-50^th^-75^th ^percentile values. Because about 3/4 of the population studied were not alcohol users on survey days, alcohol consumption levels were assigned from level 1 to 4 as 0, > 0-15, > 15-30, or > 30 grams/d (0, > 0-1, > 1-2, and > 2 servings/d). For demographic variables, age groups were assigned as 20-30, 31-40, 41-50, or > 50 y of age (group 1 to 4); body weight status was categorized from 1 to 3 as normal (BMI < 25), over weight (25≤BMI < 30), or obese (BMI ≥ 30); race consisted of non-Hispanic White (n = 4533), Black (African American, n = 1810), Hispanic (2766), or Other (275) assigned as race 1 to 4; and education levels were classified as lower than high school, high school diploma, or higher than high school (level 1 to 3).

### Estimation of dietary fructose intake

**Figure 1:**
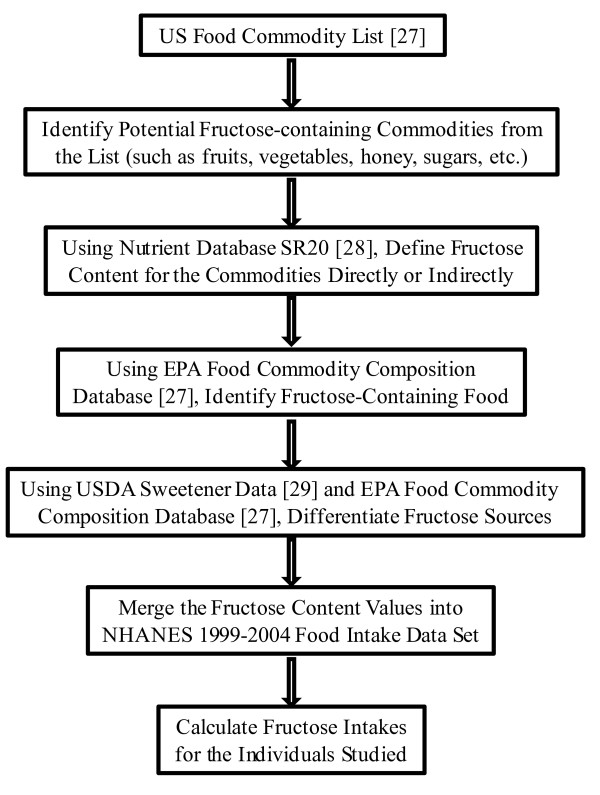
**Flow Chart for Individual Fructose Intake Calculation**.

Because of the wide distribution of fructose in foods and limitations in the available data describing its content in the majority of food items, it has been difficult to accurately calculate fructose intake for an individual. The fructose content in food items was not reported in the NHANES 1999-2004 food intake data. Years ago, Glinsmann and colleagues [26] created a specific sugar intake estimation system in which fructose was included. In this system, conversion factors were assigned for food groups based on data of added sugar disappearance and naturally-occurring sugar contents in the defined food groups. Therefore, fructose intakes could be estimated using these conversion factors and other relevant data. This method was reliable for estimating typical fructose intake in a given population (intake mean); however, this method would be less reliable to obtain data for an individual's fructose intake because of the enormous variation in fructose contents within a defined food group, such as fruits or fruit products. Thus, using this method to quantify the dietary fructose intake and its link to a health or biological outcome has limitations. For the current work, we developed a method to calculate individual fructose intakes relatively accurately (briefly summarized in the Figure 1).

In our method, the Food Commodity Intake Databases (FCID), released by the U.S. Environmental Protection Agency (EPA) in 2000 [27] and the USDA National Nutrient Database for Standard Reference (SR20), published on the website of USDA Agricultural Research Service (ARS) [28] were used to document the fructose contents for fructose-containing food commodities. In total, fructose content data of 119 from 548 commodities were obtained. If the nutrient database SR20 did not have a fructose value for a fructose-containing commodity but had the data of its family member, a ratio of the fructose to carbohydrates was used to calculate the fructose content of the commodity. For example, SR20 has the fructose content value for oranges, but not for orange juice, thus, the ratio of fructose to total carbohydrates of orange was used to indirectly calculate the fructose content value of orange juice. Accordingly, naturally-occurring unbound fructose intake of each individual can be calculated using the NHANES food intake databases, Food Commodity Composition Database, and the obtained commodity fructose content data.

With the developed method, an estimation of fructose intake from added sugars was performed using the following steps. First, added sugars were defined as dietary sugar/molasses from beet or cane, corn sweeteners (including high fructose corn syrup (HFCS)-55, HFCS-42, and corn syrup), honey, maple sugar/syrup, and sorghum syrup. These added sugars can be identified in food items using the EPA food commodity composition database. Because there are only two commodity names given to corn sweeteners (corn syrup and corn syrup for baby food), the portion of free fructose in corn sweeteners was calculated based on the ratio of HFCS-55/HFCS-42/corn-syrup of USDA 1999-2004 corn sweetener disappearance data [29]. The other unbound fructose contents in molasses/syrup/honey can be obtained from the nutrient database SR20. The bound fructose was defined as 50% of the sucrose from added sugars. Additionally, the intakes of added fructose and total fructose for individuals were calculated using the data sets as mentioned above. The reliability of this calculation method was validated by comparing the calculated fructose intake data with existing fructose intake data using the NHANES-3 and NHANES 1999-2004 database (detailed in discussion).

### Statistical analysis

SAS software (version 9.1, SAS Institute, Cary, N.C.) was utilized as the statistical evaluation tool. *P *value < 0.05 was considered as significant for statistical comparison. NHANES 2003-04 has two-day dietary data, however, NHANES 1999-2000 and 2001-02 have only one-day dietary data. In order to maintain consistency of data, we only used day-one data of NHANES 2003-04. After merging the three databases, descriptive statistics were performed with adjustment for population weight of sample size. Serum uric acid concentrations, by fructose and fiber intake quartiles, and alcohol intake levels were compared using a general linear model (GLM, with or without adjustment) with the data of the lowest intake group as reference (Dunnett control).

A logistic regression (logit) procedure was used in the risk analysis to examine potential associations of dietary fructose (added and total), alcohol, and fiber on hyperuricemia. Hyperuricemia status (yes or no) was assigned as the dependent (explained/predicted) variable of the logit models. Independent (explanatory) variables in the adjusted model included gender, age group, race, education levels, body weight status (normal, overweight or obese), dietary intake quartiles of fructose (by gram or percent of calories), energy (kcal/kJ), protein, total fat, vitamin C, fiber (grams/1000 kcal), caffeine, and alcohol intake level. The lower or lowest intake quartile, level, or category was used as reference group to obtain odds ratios for the risk of hyperuricemia. The outcomes of risk analysis (odds ratios) of fructose and fiber intake quartiles and alcohol intake level were reported because they are the focus of this work, rather than the adjusting factors. Goodness-of-fit tests were performed to recognize if the dependent variable could be satisfactorily explained by the independent variables in the models. High alcohol intake is a known factor of elevating serum uric acid concentration; beyond using it as an adjustment factor, it can also be considered as an indicator of model validation. The statistical power for testing the influence of total fructose and fiber intake on hyperuricemia risk is 87% using 2.5% as reference of hyperuricemia prevalence (actual rate was 2.57%), odds ratio changing 1/2 between intake groups, and significant threshold (α) at 0.05; the power for alcohol intake is 82% using the same baseline and odds ratio increasing 2/3 between groups.

## Results

### Subject characteristics and dietary intakes

From the databases of NHANES 1999-2004, 9,384 subjects (4,385 men and 4,999 women), between the ages of 20-80 years, without diabetes, cancer, and/or heart diseases, were included in this analysis. Table 1 summarizes subject characteristics, dietary intakes, and serum uric acid concentrations. Two hundred and forty one (241) of the 9,384 participants were identified as hyperuricemic (2.57%). Different mean values were observed between hyperuricemic and non-hyperuricemic individuals in regard to age, BMI, and intakes of energy, natural and total fructose, total sugars, carbohydrates, fiber, fruits, and alcohol. Approximately, 42% of all added sugars are fructose (bound + unbound) and added fructose can be over 80% of total fructose intake.

**Table 1:** Subject Characteristics and Dietary Intakes by Hyperuricemic Status

N = 9,384	Hyperuricemia = No, n = 9,143 (Men = 4,227, Women = 4,916)			Hyperuricemia = Yes, n = 241 (Men = 158, Women = 83)		
		
	Means	Median	SD	Means	Median	SD
Age**†**	41.51	40.00	16.21	46.77	46.00	17.40
BMI**†**	27.73	26.78	6.13	33.01	31.66	6.82
Energy (kcal/day, 1 kcal = 4.184 kJ)**†**	2317.5	2150.5	1056.6	2162.0	1979.9	950.4
Protein (g/day)	85.33	78.10	43.82	85.17	78.51	41.63
Total fat (g/day)**†**	86.54	78.02	47.44	77.91	69.49	37.98
% of energy	33.27	33.16	9.21	32.43	32.86	9.64
Saturated fat (g/day)**†**	28.57	25.28	17.07	25.80	23.11	13.63
% of energy	10.93	10.72	3.88	10.77	10.77	3.92
Carbohydrate (g/day)**†**	285.50	262.66	139.30	245.48	218.72	120.73
% of energy**†**	49.91	50.00	11.69	46.16	44.96	12.26
Total sugars (g/day)**†**	136.93	117.36	88.74	116.53	100.86	78.96
% of energy**†**	3.74	22.66	11.13	22.50	20.41	12.42
Natural unbound fructose (g/d)**†**	8.55	5.00	10.87	5.76	3.29	9.29
% of energy**†**	1.63	0.91	2.14	1.16	0.72	1.94
Added unbound fructose (g/d)	22.71	14.77	24.82	21.06	14.82	20.75
% of energy	3.81	2.65	3.71	3.98	3.23	3.74
All added fructose (g/day)**†**	42.44	33.15	34.98	37.32	30.59	29.50
% of energy	7.17	6.40	4.69	6.99	6.68	4.79
Total fructose (g/day)**†**	50.99	42.57	36.53	43.08	36.16	31.30
% of energy**†**	8.79	8.20	4.73	8.15	7.64	4.93
Fiber (g/day)**†**	15.96	13.89	10.33	12.51	11.24	9.12
Fruits/juices (g/day, n = 6580)*†	228.69	141.77	278.83	160.08	118.02	193.11
Vitamin C (mg/day)	95.23	59.55	111.97	80.58	48.20	105.06
Alcohol (g/day, n = 2506)*†	41.00	25.92	54.07	60.22	52.40	55.68
Caffeine (mg/day)*	200.60	129.00	233.12	174.42	119.35	186.84
Serum uric acid (mg/dL)**†**	5.21	5.20	1.32	8.85	8.70	0.77

**† **T-test P values < 0.05(under both of equal or unequal variance), for Vitamin C, P values = 0.067 and 0.053.

* Zero intakes are not included; serum uric acid 1 mg/dL = 59.48 μmol/L.

### Uric acid concentrations and hyperuricemia occurrence rates

The mean values of serum uric acid concentrations by fructose, alcohol, and fiber intakes are presented in Table 2. The data indicate no clear relationship between fructose intake and uric acid concentration. The higher fructose intake groups (by grams or percent of calories) had either slightly higher or slightly lower uric acid concentration means in a random manner compared to the group in the lowest intake quartile. The higher alcohol intake groups (level 3 and 4) always had significantly higher uric acid concentrations compared to the lowest intake group, with or without adjustment; while the higher fiber intake groups (quartile 2, 3 and 4) had significantly somewhat lower uric acid concentrations compared to the lowest intake group with or without adjustment adjusted. The associations of alcohol and fiber intakes with uric acid concentration also displayed a dose-dependent tendency.

**Table 2:** Serum Uric Acid Concentrations by Dietary Fructose, Alcohol, and Fiber Intakes [mean (SD), mg/dL], in Adult Population, Age 20-80 y, NHANES 1999-2004*

N = 9,384	By fructose intake quartiles				
		
	1	2	3	4	*P *of model
Added unbound fructose (g/d)	(≤ 3.84)	(≤ 14.63)	(≤ 30.04)	(>30.04)	
		
Unadjusted model	5.25 (1.48)	5.10 (1.41)^**b**^	5.23 (1.39)	5.44 (1.42)^**c**^	< 0.0001
Adjusted model	5.58 (1.56)	5.50 (1.57)^**a**^	5.58 (1.59)	5.63 (1.81)	0.004
		
All added fructose (g/d)	(≤ 15.98)	(≤ 31.60)	(≤ 54.43)	(>54.43)	
		
Unadjusted model	5.23 (1.46)	5.19 (1.43)	5.18 (1.43)	5.40 (1.40)^**c**^	0.0002
Adjusted model	5.57 (1.60)	5.57 (1.55)	5.54 (1.64)	5.59 (1.91)	0.531
		
Total fructose (g/d)	(≤ 24.29)	(≤ 41.23)	(≤ 64.11)	(>64.11)	
		
Unadjusted model	5.29 (1.49)	5.21 (1.42)	5.16 (1.41)^**b**^	5.36 (1.40)	0.01
Adjusted model	5.62 (1.62)	5.56 (1.56)	5.53 (1.65)	5.54 (1.94)	0.158

Added unbound fructose (% kcal)	(≤ 0.78)	(≤ 2.73)	(≤ 5.50)	(>5.50)	
Unadjusted model	5.28 (1.48)	5.13 (1.41)^**b**^	5.27 (1.40)	5.33 (1.42)	0.002
Adjusted model	5.58 (1.53)	5.49 (1.57)^**a**^	5.59 (1.60)	5.64 (1.76)	0.006
		
Added all fructose (% kcal)	(≤ 3.57)	(≤ 6.36)	(≤ 9.68)	(>9.68)	
		
Unadjusted model	5.35 (1.49)	5.19 (1.43)^**c**^	5.21 (1.40)^**b**^	5.26 (1.41)	0.001
Adjusted model	5.58 (1.54)	5.55 (1.54)	5.55 (1.56)	5.62 (1.84)	0.174
		
Total fructose (% kcal)	(≤ 5.48)	(≤ 8.28)	(≤ 11.48)	(>11.48)	
		
Unadjusted model	5.42 (1.50)	5.25 (1.39)^**c**^	5.14 (1.42)^**c**^	5.2 (1.41)^**c**^	< 0.0001
Adjusted model	5.58 (1.53)	5.57 (1.56)	5.53 (1.63)	5.62 (1.85)	0.098

N = 9,384	**By alcohol intake levels (g/day)**				
		
	1 (0)	2 (≤ 15)	3 (≤ 30)	4 (>30)	
	
Unadjusted model	5.12 (1.41)	5.17 (1.40)	5.73 (1.42)^**c**^	5.93 (1.39)^**c**^	< 0.0001
Adjusted model	5.31 (1.87)	5.38 (1.27)	5.65 (1.22)^**c**^	5.72 (1.39)^**c**^	< 0.0001

N = 9,384	**By fiber intake quartiles (g/1000 kcal, 1 kcal = 4.184 kJ)**				
		
	1 (≤ 4.6)	2 (≤ 6.7)	3 (≤ 9.5)	4 (>9.5)	
	
Unadjusted model	5.49 (1.50)	5.26 (1.43)^**c**^	5.18 (1.41)^**c**^	5.08 (1.36)^**c**^	< 0.0001
Adjusted model	5.60 (1.33)	5.51 (1.16)^**a**^	5.51 (1.50)^**a**^	5.45 (1.34)^**c**^	0.0016

*Serum uric acid 1 mg/dL = 59.48 μmol/L. Compared to the values of intake level 1 or quartile1, superscripted letter a represents *P *< 0.05, b represents *P *< 0.01, and c represents *P *< 0.001.

Adjusted model = adjusted for sex, age, BMI, race, education, quartile intakes of kcal, protein, total fat, total fructose (in modeling alcohol/fiber intakes), fiber/1000 kcal, and vitamin C, and alcohol intake level.

**Figure 2:**
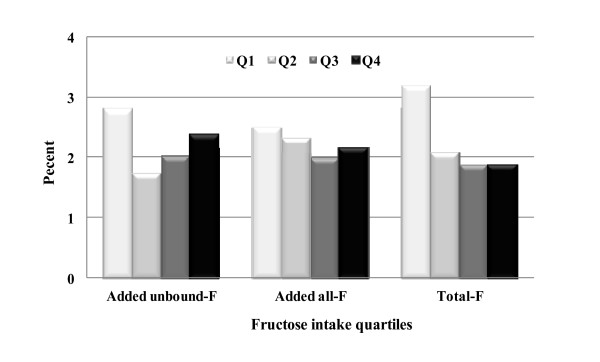
**Hyperuricemia Rates by Fructose Intakes**. F = fructose, compared to the intake quartile 1 (Q1), no statistical significance reached with the adjustment as indicated in statistical analysis.

**Figure 3:**
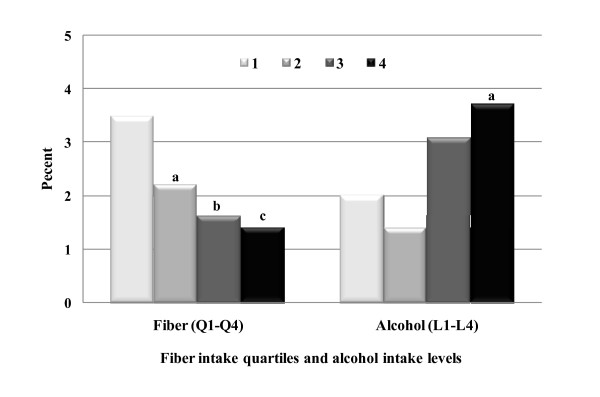
**Hyperuricemia Rates by Alcohol and Fiber Intakes**. Compared to the intake level 1 (L1) or quartile 1 (Q1), letter a = p < 0.05, b = p < 0.01 and c = p < 0.001 with the adjustment as indicated in statistical analysis. The sample Ns of alcohol intake level 1-4 are 6,836, 930, 509, and 1,109, respectively.

**Figure 4:**
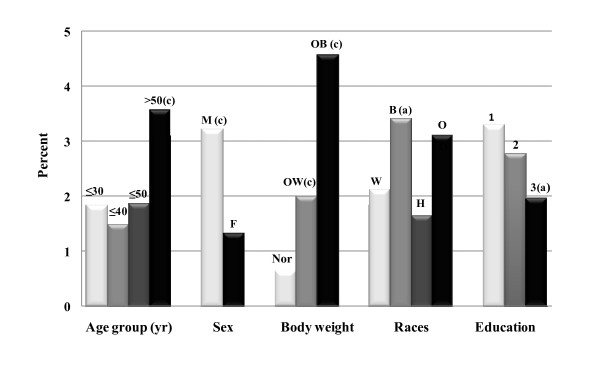
**Hyperuricemia Rates by Demographics**. For Sex, M = males (n = 4,385), F = females (n = 4,999); for Body weight, Nor = normal (n = 3,186), OW = over weight (n = 3,315), OB = obese (n = 2,893); for Races, W = Whites (n = 4,533), B = Blacks (n = 1,810), H = Hispanics (n = 2,766), O = Others (n = 275); and for education, 1 = under high school (n = 2,777), 2 = high school diploma (n = 2,223), 3 = above high school (n = 4,384). Compared to F, Age group ≤ 30, Nor, W, or education-1, letter (a) = p < 0.05, (b) = p < 0.01 and (c) = p < 0.001 with the adjustment as indicated in statistical analysis.

Figure 2 indicates hyperuricemia rates by quartiles of dietary fructose intake. No clear trend of hyperuricemia rates could be noted between quartile intake groups of fructose that came from different forms or sources, beyond subjects in quartile 1 who constantly had the highest rates. In Figure 3, subjects in higher alcohol intakes levels (3 and 4) exhibited higher hyperuricemia rates; and subjects in higher fiber intake quartiles (2, 3 and 4) had lower rates. Figure 4 depicts a clear impact of gender and body weight status, and a moderate influence of age, race, and education on hyperuricemia occurrence. Men, obese subjects, and individuals over 50 y had significantly higher rates of hyperuricemia.

### The risk of hyperuricemia

Up to 13 explanatory variables were used in the logistic regression models to explore if fructose intake was associated with the risk of hyperuricemia in the adult population. The outcomes of logistic regression on the 3 forms of fructose (added unbound, all added, and total fructose), as well as alcohol and fiber, are reported in Table 3. Odds ratios (OR (95% confidence limits)), corresponding *P *values, and results of goodness-of-fit tests are included in this table. For fructose intake, subjects in higher intake quartiles tended to have lower risk of hyperuricemia compared to the lowest intake group (all ORs < 1). Especially for total fructose intakes, a significantly lowered risk appeared in the unadjusted model in subjects with higher total fructose intakes (OR = 0.515-0.698, *P *< 0.05-0.001), tested either in grams or in % kcal. However, the noted significances did not persist in the adjusted models. Similar to previous reports, subjects in the highest level of alcohol intake were over 65% more likely to be hyperuricemic compared to those subjects in the lowest intake level (ORs = 1.658-1.829, *P *< 0.05 or < 0.001), with or without adjustment (Table 3). Furthermore, the highest fiber intake group was over 52% less likely to be hyperuricemic (ORs = 0.448-0.478, *P *< 0.001) compared to the lowest intake group, with or without adjustment. Both of these dietary factors demonstrate a dose-dependent influence on the risk of hyperuricemia. The effects of all other dietary variables examined, including total fat, protein, vitamin C, and caffeine, did not reach statistical significance, except that the higher energy intakes (quartile 3 and 4) lowered the risk mildly in the adjusted model.

**Table 3:** Influence of Fructose, Alcohol, and Fiber Intakes on the Risk of Hyperuricemia in Adult Population Aged 20-80 y, NHANES 1999-2004, Odds Ratios (95% confidence limits)*

N = 9,384	Fructose intake quartiles				
		
	1	2	3	4	*P *of model
Added unbound fructose (g/d)	(≤ 3.84)	(≤ 14.63)	(≤ 30.04)	(>30.04)	
		
Unadjusted model	1	0.625 (0.435-0.899)^**a**^	0.717 (0.506-1.016)	0.758 (0.537-1.069)	0.061
Adjusted model	1	0.731 (0.501-1.066)	0.815 (0.554-1.197)	0.916 (0.586-1.431)	0.381
		
All added fructose (g/d)	(≤ 15.98)	(≤ 31.60)	(≤ 54.43)	(>54.43)	
		
Unadjusted model	1	0.823 (0.583-1.163)	0.716 (0.501-1.025)	0.728 (0.51-1.04)	0.215
Adjusted model	1	0.952 (0.659-1.375)	0.896 (0.589-1.363)	0.992 (0.598-1.645)	0.944
		
Total fructose (g/d)	(≤ 24.29)	(≤ 41.23)	(≤ 64.11)	(>64.11)	
		
Unadjusted model	1	0.642 (0.457-0.901)^**a**^	0.576 (0.405-0.817)^**b**^	0.515 (0.359-0.74)^**c**^	0.001
Adjusted model	1	0.694 (0.481-1.001)	0.697 (0.458-1.06)	0.603 (0.357-1.019)	0.154

Added unbound fructose (% kcal)	(≤ 0.78)	(≤ 2.73)	(≤ 5.50)	(>5.50)	
		
Unadjusted model	1	0.622 (0.432-0.897)^**a**^	0.727 (0.512-1.032)	0.807 (0.573-1.136)	0.068
Adjusted model	1	0.736 (0.504-1.075)	0.831 (0.567-1.217)	0.854 (0.564-1.293)	0.456
		
Added all fructose (% kcal)	(≤ 3.57)	(≤ 6.36)	(≤ 9.68)	(>9.68)	
		
Unadjusted model	1	0.663 (0.464-0.947)	0.734 (0.518-1.039)	0.716 (0.504-1.016)	0.088
Adjusted model	1	0.785 (0.54-1.14)	0.868 (0.59-1.275)	0.766 (0.489-1.2)	0.549
		
Total fructose (% kcal)	(≤ 5.48)	(≤ 8.28)	(≤ 11.48)	(>11.48)	
		
Unadjusted model	1	0.644 (0.453-0.915)^**a**^	0.698 (0.495-0.984)^**a**^	0.617 (0.432-0.882)^**b**^	0.02
Adjusted model	1	0.754 (0.521-1.091)	0.853 (0.578-1.259)	0.715 (0.453-1.128)	0.374

N = 9384	**Alcohol intake levels (g/d)**				
		
	1 (0)	2 (≤ 15)	3 (≤ 30)	4 (>30)	
	
Unadjusted model	1	0.787 (0.475-1.304)	1.729 (1.076-2.777)^**a**^	1.829 (1.309-2.556)^**c**^	< 0.001
Adjusted model	1	0.86 (0.513-1.443)	1.511 (0.912-2.505)	1.658 (1.078-2.549)^**a**^	0.057

N = 9384	**Fiber intake quartiles (g/1000 kcal, 1 kcal = 4.184 kJ)**				
		
	1 (≤ 4.6)	2 (≤ 6.7)	3 (≤ 9.5)	4 (>9.5)	

Unadjusted model	1	0.599 (0.427-0.839)^**b**^	0.522 (0.367-0.742)^**c**^	0.478 (0.333-0.686)^**c**^	< 0.001
Adjusted model	1	0.637 (0.445-0.912)^**a**^	0.552 (0.373-0.816)^**b**^	0.448 (0.291-0.69)^**c**^	0.0013

*Compared to the values of intake quartile1 or level 1, superscripted letter a represents *P *< 0.05, b represents *P *< 0.01, and c represents *P *< 0.001.

Adjusted model = adjusted for sex, age, BMI, race, education, quartile intakes of kcal, protein, total fat, total fructose (in modeling alcohol/fiber intakes), fiber/1000 kcal, and vitamin C, and alcohol intake level.

The range of *P *values for tests of model Goodness of Fit was between 0.17-0.87, which indicates that dependent variable could be satisfactorily explained by independent variables.

Among the demographic variables, gender, age, race, body weight status, and education level all had a significant influence on hyperuricemia risk (Figure 4). Women were about 58% less likely to be hyperuricemic than men were, OR = 0.418 (95% CI, 0.31-0.563). Adults over 50 y were more than 100% more likely to be hyperuricemic compared to people ≤ 30 y, OR = 2.007 (1.35-2.984). Compared to non-Hispanic Whites, Black adults had about 50% higher chance of being hyperuricemic, OR = 1.506 (1.077-2.107). Unexpectedly, obese adults were at least 3-fold, and over-weight adults were at least 1-fold, more likely to be hyperuricemic than adults with BMI values below 25, OR = 4.81 (3.214-7.198) or 2.445 (1.611-3.712), respectively. Additionally, adults with the highest education level (above high school diploma) seemed to be at lower risk than adults with the lowest education level (under high school), OR = 0.644 (0.46-0.901). For simplification, the detailed outcomes of logistic regression of these variables are not reported.

To confirm the validity of the statistical model used, goodness-of-fit tests were performed. The large *P *values (0.17-0.87) of goodness-of-fit tests for adjusted models indicated that the dependent variable (hyperuricemia yes or no) could be satisfactorily explained by the independent variables.

## Discussion

In this study, dietary fructose intake was examined for a potential association with the risk of hyperuricemia in adults, using the data contained in NHANES 1999-2004. This work is the first effort to evaluate possible associations of various factors, particularly fructose, with the risk of hyperuricemia using multiple U.S. national nutrition survey databases. Dietary intakes of fructose were not found to be associated with a higher risk of hyperuricemia, while alcohol and fiber had significant influence on the risk in opposite directions. Intakes of protein, fat, vitamin C, and caffeine had no significant influence on the risk. Unexpectedly, subjects in the highest total fructose intake group (by gram or % kcal) tended to have lower hyperuricemia risk (OR = 0.515-0.715); however, the data are not strong enough to suggest that the higher total fructose intake may be linked with lower risk of hyperuricemia. Differences in the serum uric acid concentrations between the fructose intake quartiles were very small (all under 5%). Some concentration means in the groups of quartile 2, 3, or 4 appeared to be significantly lower or higher in a random manner compared to the lowest intake group (Table 2), and the observed concentration changes were not consistent with the outcomes from risk analysis. This result suggests that clinical significance of the observed changes in serum uric acid concentration is not of importance.

Previously, Gao and colleagues [30] reported the influence of added sugar and sugar-sweetened drink intakes (containing both glucose and fructose) on the concentrations of serum uric acid in the adult population from NHANES 2001-02 database, but this work did not examine relationships specific to fructose intake. Based on the different model conditions, subjects in the highest sugar intake quartile had 3.8-6.6% elevated serum uric acid concentration compared to the data of subjects in the lowest sugar intake quartile. Those increases of uric acid concentration reached statistical significance. This outcome appeared to be comparable to our data. However, our results from a larger sample size did not produce an appreciable statistical significance for added fructose, either with or without adjustments. Gao et al. did not report whether the slight increase of serum uric acid was linked with any clinical outcomes. Additionally, as noted by both Gao's work and ours, the means of uric acid concentrations in different intake quartiles of total sugar or fructose were all near the middle value of the normal range. Hence, these observations indicate that concentration alone might not be enough to explain a potential influence of dietary sugars on serum uric acid. Gao et al also reported that higher vitamin C intake was associated with lower serum uric acid concentration in 1,387 non-obese men [31]. In the current work, it was noted as well that hyperuricemic subjects had an obviously lower vitamin C intake mean than that of non-hyperuricemic adults (80.6 to 95.2 mg/d, Table 1). However, it was not seen that higher vitamin C intake was associated with lower hyperuricemia risk.

The influence of fructose intake on risk of hyperuricemia was reported by Choi et al. [32] using older NHANES-3 (1988-94) data. Choi and colleagues selected a cohort age over 50 y; grouped them by fructose intakes of < 10, 10-49.9, 50-74.9, and ≥ 75 g/d; and reported a significant trend between odds ratios and corresponding fructose consumption levels without reporting comparisons between groups and 95% confidence limits of odds ratios. Because of the differences in study subjects, categorization of fructose intake groups, and criteria of defining hyperuricemia, it is less meaningful to compare our fructose intake outcomes with Choi's report.

Previously, the cut-off values >7 and >6 mg/dl of uric acid concentration were used for defining hyperuricemia in certain studies. To check whether the different definition altered our results, we ran the statistical analysis using the lower cut-off reference values to define hyperuricemia for men and women, respectively. The results obtained are very similar between the two sets of cut-off values used to define hyperuricemia, except that prevalence rate was increased to 15.14% for the lower cut-off compared to 2.57% for the cut-off used in the current work. Table 4 shows descriptive statistics of hyperuricemia prevalence by levels of fructose, alcohol and fiber intakes using the >7 and >6 mg/dl cut-offs. This outcome is not unexpected. Logistic regression analysis also indicated: 1) no hyperuricemia risk increase in the higher fructose intake groups, 2) significant risk increase in the highest alcohol intake group, and 3) significant risk decrease in the highest fiber intake group, compared to the other intake ends. Consequently, our conclusions are not changed by using either of the two sets of cut-off points to define hyperuricemia.

**Table 4:** Hyperuricemia rates (%) by fructose, alcohol, and fiber intakes*

Fructose intakes	Intake quartiles				Intake quartiles, Weighted data			
		
	1	2	3	4	1	2	3	4
Added all fructose (g/d)	16.07	16.09	13.61	14.8	15.69	16.02	14.2	15.79
All fructose (g/d)	18.15	15.22	12.94	14.27	17.65	14.91	13.62	15.58
Added all fructose (%E)	17.21	14.71	13.25	15.42	16.47	15.6	13.37	16.24
All fructose (%E)	18.23	14.19	12.99	15.16	18.09	14.2	13.56	15.66

**Alcohol and fiber intakes**	**Intake level or quartile**				**Intake level or quartile, Weighted data**			
		
	**1**	**2**	**3**	**4**	**1**	**2**	**3**	**4**

Alcohol	13.82	12.47	19.84	23.35	14.49	11.31	17.81	22.25
Fiber (g/1000 kcal)	19.1	15.31	14.24	11.97	18.98	16.33	14.04	11.71

* Using uric acid concentration cut-off values, >7 and >6 mg/dl, as definitions of hyperuricemia for men and women, respectively

Human experimental or interventional studies exploring the relationship between fructose administration and serum uric acid concentration produced inconsistent results. MacDonald and colleagues noted that serum uric acid concentration increased about 10% within 90 minutes after a pure fructose drink (1 g/kg BW) in nine healthy young men [10], while Emmerson indicated that intakes of 250-290 g/d fructose increased serum uric acid concentration 8-41% compared to glucose in three healthy men [11]. Two fructose infusion studies (0.5 g/kg BW, in 8 healthy young men and 4 gout patients) also observed an increase of serum uric acid levels [12,22]. However, other studies did not observe increases in serum uric acid concentration from fructose administration. In the Finland Turku sugar studies, 35 healthy subjects continuously ingested added fructose at a level of 2.1 kg/month (approximately 70 g/d) for 22 months and had no appreciable increase in serum uric acid concentration or uric acid excretion [14]. Crapo et al [13] observed that 2-week fructose administration at 24% of daily calorie did not raise serum uric acid concentration and urinary excretion in 11 normal subjects. Narins and colleagues reported that 5-d added fructose intake at 100 g/d did not cause an increase of serum uric acid concentration in healthy subjects [22]. Also, Turner and colleagues reported that serum uric acid was not elevated after drinking a beverage containing 90-154 g fructose in 6 hypertriglyceridemic men [15]. Likewise, the study by Curreri showed that infusion of 100 g fructose did not induce serum uric acid increases in 20 young men [16]. Moreover, Osei [17,18], Anderson [19], Koh [20], and Grigoresco [21] all reported that long-term (1 to 6 months) intake of added fructose at 11-18% of daily calories did not influenced serum uric acid concentrations in diabetic subjects. A possible explanation for the observed inconsistencies in these intervention trials may be differences in subjects and study protocols. Another important point is that fructose administered alone may produce very different absorption, metabolism and physiological effects compared to its co-ingestion with other carbohydrates, such as glucose. A glucose co-effect was documented by Riby et al concerning fructose mal-absorption [33]. In ordinary diets, fructose is rarely consumed alone. We also noted in this work that dietary fructose intake was allied with other sugars as indicated by Pearson and Spearman correlations (both r = 0.94, *P *< 0.0001, between intakes of fructose and total sugars).

In agreement with previous reports, high alcohol consumption (especially more than 30 g/d alcohol or 2 drinks/d) increased the risk of hyperuricemia by more than 65% compared to adults who did not consume alcohol (Table 3). There are a number of publications that have investigated the links between alcohol consumption and serum uric acid concentration. A proposed mechanism for dietary alcohol inducing serum uric acid increase is related to high purine contents in alcoholic beverages (beer) and to alcohol metabolism which unusually causes adenosine triphosphate break down to purines, as reviewed by Yamamoto [34]. The agreement of our work and previous studies on alcohol consumption and uric acid lends credence to the validity of the logit model used herein.

In contrast, we did not find previously published literature examining the relationship specifically between dietary fiber intake and hyperuricemia risk in the general adult population. A possible mechanism of higher fiber intake associated with lower serum uric acid concentration and reduced hyperuricemia risk could be that dietary fiber inhibits purine or adenine absorption in the digestive system [35]. Also, higher fiber intakes are usually associated with healthier diets (higher fruit and vegetable intake) and lifestyle.

Beyond dietary and demographic factors, other aspects may be more important in controlling serum uric acid concentration. In a review by Luk and Simkin [36], it was noted that inefficient excretion of uric acid, which accounts for >90% of hyperuricemic cases, can be the result of renal insufficiency of any cause that impairs renal urate clearance. It was also reported that the kidney plays a dominant role in maintaining plasma urate levels through the excretion process, and the normal functioning of several urate efflux transporters in kidney is critical for facilitating the excretion process [37,38]. Thus, normal kidney urate clearance performs a more deciding role than dietary factors in hyperuricemia development.

A novelty and possible weakness of this work is the calculation method used to determine total fructose intake. Due to the lack of fructose content data for many food items in the databases, this work established a method to estimate fructose contents in added sugars and a method to estimate naturally-occurring fructose contents in foods. Because of a lack of data for naturally-occurring sucrose contents for most foods, the naturally-occurring bound fructose from sucrose was not available for analyzing individual intake. However, after careful evaluation, we realized that the amount of underestimated bound fructose from natural sucrose was small and would be evenly distributed in the subjects of each fructose intake quartiles. In the aggregate population studied, the mean intakes of total sugars, added sugars, and lactose were 135.91, 99.86, and 14.49 g/d per person, respectively. After subtracting the added and dairy sugars from total sugar intakes, the remainder would be natural sugars in foods. Most of them, if not all, would be from fruits or vegetables. Thus, based on the intake data of natural sucrose and total natural sugars from fruits and vegetables reported by USDA ERS [39], a ratio of sucrose to total sugars was calculated as 26.53% (a mean from 1990-2001 data). The underestimated naturally-occurring bound fructose was further calculated: (135.91- 99.86 - 14.49) * 26.53% * 1/2 = 2.86 grams, at the maximum, which is about 5.6% of total fructose intake of the current study. Recently, Marriott and colleagues [40] reported estimates of typical fructose intake data in U.S. population using food group-specific fructose conversion factors and the same databases (NHANES 1999-2004). The total fructose intake mean in the adults (age ≥ 19 y) is 44.5 g/day (recalculated based on the reported data by age group) using an unweighted sample size. By using an unweighted sample size, our estimate of the total fructose intake mean is 43.1 g/day in the adults (age 20-80 y). The values from the two different methods are in close agreement. Moreover, Vos et al. [41] reported that mean of total fructose intake was 51.8 g/d (recalculated based on reported data) in adults with age ≥ 19 y from an analysis of the NHANES-3 database (weighted sample size used). This estimate was also similar with the result of our study (50.53 g/d, using weighted sample size). However, the NHANES-3 data were collected between 1988-1994, about 10 years earlier than the data used in our work. Based on the assessments mentioned above, this newly developed method for estimating individual fructose intake should be reliable.

In conclusion, higher dietary fructose intake was not associated with increased risk of hyperuricemia among adult participants in the NHANES 1999-2004; higher dietary alcohol intake was associated with increased risk; higher dietary fiber intake was associated with decreased risk; and older individuals, males, or the obese all had greater risk. Fructose administration under experimental conditions (by acute high-dose ingestion or intravenous infusion) may produce outcomes that are variable and incomparable with those from fructose in ordinary diets containing other sugars when relating serum uric acid status. Beyond dietary factors, the impact of demographic factors on serum uric acid concentration and hyperuricemia occurrence continues to merit attention.

## Competing interests

This research was conducted by Archer Daniels Midland Company (ADM). The authors are employed full time by ADM. ADM is a major oilseed and grain commodity processor and produces, among other products, fructose-containing sweeteners, alcohol and fiber.

## Authors' contributions

All authors contributed equally to the study design and manuscript writing. Dr. Sam Z. Sun conducted the data extraction and statistical analysis. All authors read and approved the final manuscript.

## References

[B1] Johnson RJ Segal MS Sautin Y Nakagawa T Feig DI Kang DH Gersch MS Benner S Sanchez-Lozada LG Potential role of sugar (fructose) in the epidemic of hypertension, obesity and the metabolic syndrome, diabetes, kidney disease, and cardiovascular disease Am J Clin Nutr 2007 86 4 899 906 17921363 10.1093/ajcn/86.4.899

[B2] Kawamoto R Tomita H Oka Y Ohtsuka N Relationship between serum uric acid concentration, metabolic syndrome and carotid atherosclerosis Intern Med 2006 45 9 605 614 10.2169/internalmedicine.45.1661 16755091

[B3] Choi HK Ford ES Prevalence of the metabolic syndrome in individuals with hyperuricemia Am J Med 2007 120 5 442 447 10.1016/j.amjmed.2006.06.040 17466656

[B4] Heinig M Johnson RJ Role of uric acid in hypertension, renal disease, and metabolic syndrome Cleve Clin J Med 2006 73 12 1059 1064 10.3949/ccjm.73.12.1059 17190309

[B5] Nakagawa T Tuttle KR Short RA Johnson RJ Hypothesis: fructose-induced hyperuricemia as a causal mechanism for the epidemic of the metabolic syndrome Nat Clin Pract Nephrol 2005 1 2 80 86 10.1038/ncpneph0019 16932373

[B6] Yoo TW Sung KC Shin HS Kim BJ Kim BS Kang JH Lee MH Park JR Kim H Rhee EJ Lee WY Kim SW Ryn SH Keum DG Relationship between serum uric acid concentration and insulin resistance and metabolic syndrome Circ J 2005 69 8 928 933 10.1253/circj.69.928 16041161

[B7] Chien KL Chen MF Hsu HC Chang WT Su TC Lee YT Hu FB Plasma uric acid and the risk of type 2 diabetes in a Chinese community Clin Chem 2008 54 2 310 316 10.1373/clinchem.2007.095190 18089655

[B8] Nan H Qiao Q Soderberg S Gao W Zimmet P Shaw J Alberti G Dong Y Uusitalo U Pauvaday V Chitson P Tuomilehto J Serum uric acid and components of the metabolic syndrome in non-diabetic populations in Mauritian Indians and Creoles and in Chinese in Qingdao, China Metab Syndr Relat Disord 2008 6 1 47 57 10.1089/met.2007.0028 18370836

[B9] Johnson RJ Perez-Pozo SE Sautin YY Manitius J Sanchez-Lozada LG Feig DI Shafiu M Segal M Glassock RJ Shimada M Roncal C Nakagawa T Hypothesis: could excessive fructose intake and uric acid cause type 2 diabetes? Endocr Rev 2009 30 1 96 116 10.1210/er.2008-0033 19151107 PMC2647706

[B10] Macdonald I Keyser A Pacy D Some effects, in man, of varying the load of glucose, sucrose, fructose, or sorbitol on various metabolites in blood Am J Clin Nutr 1978 31 8 1305 1311 677070 10.1093/ajcn/31.8.1305

[B11] Emmerson BT Effect of oral fructose on urate production Ann Rheum Dis 1974 33 3 276 280 10.1136/ard.33.3.276 4843132 PMC1006256

[B12] Fox IH Kelley WN Studies on the mechanism of fructose-induced hyperuricemia in man Metabolism 1972 21 713 721 10.1016/0026-0495(72)90120-5 5047915

[B13] Crapo PA Kolterman OG The metabolic effects of 2-week fructose feeding in normal subjects Am J Clin Nutr 1984 39 4 525 534 6369956 10.1093/ajcn/39.4.525

[B14] Huttunen JK Makinen KK Scheinin A Turku sugar studies XI. Effects of sucrose, fructose and xylitol diets on glucose, lipid and urate metabolism Acta Odontol Scand 1976 34 6 345 351 10.3109/00016357609004646 1070904

[B15] Turner JL Bierman EL Brunzell JD Chait A Effect of dietary fructose on triglyceride transport and glucoregulatory hormones in hypertriglyceridemic men Am J Clin Nutr 1979 32 5 1043 1050 433820 10.1093/ajcn/32.5.1043

[B16] Curreri PW Pruitt BA Jr Absence of fructose-induced hyperuricaemia in men Lancet 1970 1 7651 839 10.1016/S0140-6736(70)92436-0 4191459

[B17] Osei K Bossetti B Dietary fructose as a natural sweetener in poorly controlled type 2 diabetes: a 12-month crossover study of effects on glucose, lipoprotein and apolipoprotein metabolism Diabet Med 1989 6 6 506 511 10.1111/j.1464-5491.1989.tb01218.x 2527132

[B18] Osei K Falko J Bossetti BM Holland GC Metabolic effects of fructose as a natural sweetener in the physiologic meals of ambulatory obese patients with type II diabetes Am J Med 1987 83 2 249 255 10.1016/0002-9343(87)90693-0 3618627

[B19] Anderson JW Story LJ Zettwoch NC Gustafson NJ Jefferson BS Metabolic effects of fructose supplementation in diabetic individuals Diabetes Care 1989 12 5 337 344 10.2337/diacare.12.5.337 2721342

[B20] Koh ET Ard NF Mendoza F Effects of fructose feeding on blood parameters and blood pressure in impaired glucose-tolerant subjects J Am Diet Assoc 1988 88 8 932 938 3294273

[B21] Grigoresco C Rizkalla SW Halfon P Bornet F Fontvieille AM Bros M Dauchy F Tchobroutsky G Slama G Lack of detectable deleterious effects on metabolic control of daily fructose ingestion for 2 mo in NIDDM patients Diabetes Care 1988 11 7 546 550 10.2337/diacare.11.7.546 3203571

[B22] Narins RG Weisberg JS Myers AR Effects of carbohydrates on uric acid metabolism Metabolism 1974 23 5 455 465 10.1016/0026-0495(74)90093-6 4825302

[B23] Livesey G Fructose ingestion: dose-dependent responses in health research J Nutr 2009 139 6 1246S 1252S 10.3945/jn.108.097949 19386821

[B24] Schlesinger N Dietary factors and hyperuricaemia Curr Pharm Des 2005 11 32 4133 4138 10.2174/138161205774913273 16375734

[B25] CDC NHANES 2003-04 Laboratory Procedure Manual http://www.cdc.gov/nchs/data/nhanes/nhanes_03_04/l40_c_met_uric_acid.pdf

[B26] Glinsmann WH Irausquin H Park YK Evaluation of health aspects of sugars contained in carbohydrate sweeteners. Report of Sugars Task Force, 1986 J Nutr 1986 116 11 Suppl S1 216 3543257 10.1093/jn/116.suppl_11.S1

[B27] The U.S. Environmental Protection Agency (EPA), Division CEBHE Food Commodity Intake Database (FCID), CD-ROM 2000 http://www.ntis.gov/search/index.aspx

[B28] (ARS) UARS The USDA National Nutrient Database for Standard Reference (SR20) 2008 http://www.nal.usda.gov/fnic/foodcomp/search/

[B29] USDA ERS Table 30--U.S. high fructose corn syrup (HFCS) supply and use 2008 http://www.ers.usda.gov/Briefing/Sugar/Data.htm

[B30] Gao X Qi L Qiao N Choi HK Curhan G Tucker KL Ascherio A Intake of added sugar and sugar-sweetened drink and serum uric acid concentration in US men and women Hypertension 2007 50 2 306 312 10.1161/HYPERTENSIONAHA.107.091041 17592072

[B31] Gao X Curhan G Forman JP Ascherio A Choi HK Vitamin C intake and serum uric acid concentration in men J Rheumatol 2008 35 9 1853 1858 18464304 PMC2853937

[B32] Choi JW Ford ES Gao X Choi HK Sugar-sweetened soft drinks, diet soft drinks, and serum uric acid level: the Third National Health and Nutrition Examination Survey Arthritis Rheum 2008 59 1 109 116 10.1002/art.23245 18163396

[B33] Riby JE Fujisawa T Kretchmer N Fructose absorption Am J Clin Nutr 1993 58 5 Suppl 748S 753S 8213606 10.1093/ajcn/58.5.748S

[B34] Yamamoto T Moriwaki Y Takahashi S Effect of ethanol on metabolism of purine bases (hypoxanthine, xanthine, and uric acid) Clin Chim Acta 2005 356 1-2 35 57 10.1016/j.cccn.2005.01.024 15936302

[B35] Koguchi T Koguchi H Nakajima H Takano S Yamamoto Y Innami S Maekawa A Tadokoro T Dietary fiber suppresses elevation of uric acid and urea nitrogen concentrations in serum of rats with renal dysfunction induced by dietary adenine Int J Vitam Nutr Res 2004 74 4 253 263 10.1024/0300-9831.74.4.253 15580807

[B36] Luk AJ Simkin PA Epidemiology of hyperuricemia and gout Am J Manag Care 2005 11 15 Suppl S435 442 16300457

[B37] Caulfield MJ Munroe PB O'Neill D Witkowska K Charchar FJ Doblado M Evans S Eyheramendy S Onipinla A Howard P Shaw-Hawkins S Dobson RJ Wallace C Newhouse SJ Brown M Connell JM Dominiczak A Farrall M Lathrop GM Samani NJ Kumari M Marmot M Brunner E Chambers J Elliott P Kooner J Laan M Org E Veldre G Viigimaa M Cappuccio FP Ji C Iacone R Strazzullo P Moley KH Cheeseman C SLC2A9 is a high-capacity urate transporter in humans PLoS Med 2008 5 10 e197 10.1371/journal.pmed.0050197 18842065 PMC2561076

[B38] Anzai N Ichida K Jutabha P Kimura T Babu E Jin CJ Srivastava S Kitamura K Hisatome I Endou H Plasma urate level is directly regulated by a voltage-driven urate efflux transporter URATv1 (SLC2A9) in humans J Biol Chem 2008 283 40 26834 26838 10.1074/jbc.C800156200 18701466

[B39] USDA ERS Natural fructose intake levels from some fruits and vegetables (g/day person), data sheet 1970-2001 2003 http://www.ers.usda.gov/Data/

[B40] Marriott BP Cole N Lee E National estimates of dietary fructose intake increased from 1977 to 2004 in the United States J Nutr 2009 139 6 1228S 1235S 10.3945/jn.108.098277 19403716

[B41] Vos MB Kimmons JE Gillespie C Welsh J Blanck HM Dietary fructose consumption among US children and adults: the Third National Health and Nutrition Examination Survey Medscape J Med 2008 10 7 160 18769702 PMC2525476

